# Predictive value of ACEF score for clinical prognosis of patients with heavily calcified coronary lesions after percutaneous coronary intervention with rotational atherectomy

**DOI:** 10.1186/s13019-022-01833-7

**Published:** 2022-04-27

**Authors:** Hongwu Chen, Xiaofan Yu, Guangquan Qiu, Likun Ma

**Affiliations:** Department of Cardiology, The First Affiliated Hospital of USTC (Anhui Provincial Hospital), Division of Life Sciences and Medicine, University of Science and Technology of China, No. 17 Lujiang Road, Luyang District, Hefei, 230001 Anhui Province China

**Keywords:** ACEF, Heavily calcified coronary lesions, Rotational atherectomy, Prognosis

## Abstract

**Background:**

To inquiry the predictive value of the age, creatinine, and ejection fraction (ACEF) score for cardiac mortality in patients diagnosed with heavily calcified coronary lesions at 1 year after percutaneous coronary intervention (PCI) with rotational atherectomy (RA).

**Methods:**

275 patients with heavily calcified coronary lesions undergoing PCI with RA in the Department of Cardiology of Anhui Provincial Hospital from January 2017 to December 2019 were consecutively recruited. The primary endpoint event was cardiac death at postoperative 1 year. The ROC curve was used to assess ACEF scoring system and predict cardiac mortality.

**Results:**

In term of ACEF score upon admission, 275 patients were divided into low-to-intermediate risk group (n = 130) with ACEF score < 1.23 and high-risk group (n = 145) with ACEF score ≥ 1.23. The age, gender proportion and left ventricular ejection fraction (LVEF) have a significant difference between the low-to-intermediate risk group and the high-risk group (all *P* < 0.05). The area under ROC curve for ACEF scoring system to predict cardiac mortality at 1 year after PCI with RA was 0.756 and 0.715, respectively.

**Conclusions:**

ACEF value upon admission can predict the cardiac mortality at 1 year following PCI with RA in heavily calcified coronary lesions patients.

## Background

Percutaneous coronary intervention (PCI) is one of the most important and effective treatment methods for coronary heart disease. Furthermore, with the advancement of PCI equipment and technology, more complicated coronary artery diseases have been successfully treated. However, there are still some special coronary artery diseases that cannot be completely overcome including heavily calcified coronary lesions. Heavily calcified coronary lesions can limit the effective pre-dilation of the balloon, increase the risk of complications such as coronary artery tearing, vascular dissection, difficulty in passing stents, perforation, poor stent attachment and no reflow, reduce the success rate of surgery, cause postoperative restenosis and thrombosis in the stent, and affect the long-term curative effect. Therefore, PCI treatment of heavily calcified coronary lesions is full of challenges [1]. Gratifyingly, lesion processing by an atherectomy strategy prior to PCI is an effective means for heavily calcified coronary lesions [2].

Recent studies have shown that rotational atherectomy (RA) with the Rotablator (Boston Scientific, Natick, MA, USA) contributes to treat heavily calcified coronary lesions. RA operate in a drill-like way, generating a maximum burr to vessel ratio of 0.7 [3]. RA can help stent release and adherence to the wall, improve the success rate of interventional therapy for severely calcified lesions, reduce the incidence of in-stent restenosis and in-stent thrombosis, and improve clinical prognosis. Therefore, according to the 2011 PCI guidelines, RA is recommended to treat the heavily calcified lesions or fibrotic that is difficult to penetrate or fully expand before stent placement (Class IIA recommendation) [4]. However, it is still very necessary and important to evaluate the risks and benefits (known as the risk stratification analysis) of RA on the patients with heavily calcified coronary lesions [5].

The ACEF score is a clinical risk score comprising age, creatinine, and left ventricular ejection fraction (LVEF) [6]. Although the ACEF score is originally proposed and validated in patients before undergoing coronary artery bypass grafting [7], it has been demonstrated to be applicable for PCI patients through multiple clinical trials [8, 9]. Our recent study has also shown that the ACEF value upon admission can predict the cardiac death rate at 1 month and 1 year after emergency PCI in STEMI patients aged ≥ 75 years old [10]. Furthermore, Pyxaras et al*.* [11] has exhibited that the ACEF score can predict major adverse cardiovascular events (MACE) in patients diagnosed with heavily calcified coronary stenosis at 1 year after RA with stent implantation. However, whether ACEF score can be utilized to predict the cardiac mortality in Chinese patients diagnosed with heavily calcified coronary lesions at 1 year after PCI with RA still need further reports.

In the present study, 275 patients with heavily calcified coronary lesions undergoing PCI with RA in the Department of Cardiology of Anhui Provincial Hospital from January 2017 to December 2019 were consecutively recruited to elucidate the predictive value of ACEF score for the cardiac death at 1 year after PCI with RA.

## Methods

### Study population

Patients with heavily calcified coronary lesions undergoing PCI with RA in the Department of Cardiology of Anhui Provincial Hospital from January 2017 to December 2019 were consecutively recruited in this clinical trial. Any of the following conditions were defined as heavily calcified coronary lesions: (1) Coronary angiography showed a clear and high-density image walking along the blood vessel wall, and it could also be appeared after the contrast agent is filled and the heart is not beating [12]. (2) Intravascular ultrasound showed that the hyperechoic light clusters distributed along the blood vessel wall are accompanied by sound shadows, and the lesions was with a range of ≥ 181° [13]. The study was performed in accordance with the Declaration of Helsinki (as revised in 2013). The study was approved by the Medical Ethics Committee of Anhui Provincial Hospital [Ethical approval number: 2019 KY Lun Shen No. 165] and informed consent was taken from all the patients.

### Examinations and parameters

The examinations and parameters used in the present study were in accordance with our recent study [10].

### Procedural Protocol

Rotablator III rotational atherectomy device (model H802220200381) was used for coronary RA. Rotatheter guide wire, head and propeller were adopted Rota WireTM (0.09 in × 330 cm, 1 in = 2.54 cm) (Boston Scientific, USA), Rota Link M (diameters are 1.25 mm, 1.50 mm and 1.75 mm) (Boston Scientific, USA), and Rota Link M (Boston Scientific, USA), respectively. Rotary atherectomy washing solution [saline 500 ml, nitroglycerin 2 mg, heparin 5000 U; pressure 150–200 mmHg (1 mmHg = 0.133 kPa)] was used for pressure perfusion. Before the start of RA, patients were received heparin with a dosage of 60–100 U/kg, and then added 1000–2000 U/h to maintain activated coagulation time (ACT) > 300 s. Before and after coronary RA, nitroglycerin with a dosage of 50–200 μg was given in the coronary arteries as appropriate. The RA was executed at a rotation speed of 14–18 × 10^4^ r/min, with a rotation time of each rotary grinding of 15–30 s, and with a time interval of 30–120 s. The procedural protocol of coronary angiography used in the present study were in accordance with our recent study [10].

### ACEF scoring analysis

The ACEF score was analyzed based on the following formula: ACEF = age/left ventricular ejection fraction (%) + 1 (if creatinine was > 2.0 mg/dL). All patients were divided into tertiles according to the ACEF score [14].

### Primary and secondary endpoint events

The primary endpoint event of the study was cardiac mortality within postoperative 1 year. Secondary endpoint events contained death (cardiogenic and non-cardiogenic), bleeding (BARC classification), stent thrombosis, recurrent myocardial infarction, complete revascularization, stent restenosis (ISR), and cerebral infarction within 1 year after PCI with RA. All patients were followed up by outpatient, medical record or telephone call. The follow-up time was 1 year or until the end-point event occurred.

### Statistical analysis

Data in this present study were analyzed using SPSS 20.0 statistical software. All the measurement data in the present study were expressed as mean ± standard deviation (SD) and analyzed with the *t*-test, while all the count data were expressed as percentage (%) and analyzed by the chi-square test using SPSS 20.0 statistical software (SPSS Inc., Chicago, IL, U.S.). The ROC curve was used to evaluate the ACEF scoring system to predict the postoperative 1-year mortality rate. Cox regression model was executed using univariate analysis. *P* < 0.05 was indicated as statistically significant.

## Results

### Patient grouping

275 patients with heavily calcified coronary lesions undergoing PCI with RA from January 2017 to December 2019 in the Department of Cardiology of Anhui Provincial Hospital were consecutively recruited in this clinical trial. There were 163 male and 112 female patients respectively. Based on the ACEF score upon admission, all patients were divided into the low-to-intermediate risk group (n = 130) and high-risk group (n = 145). In the low-to-intermediate risk group, there were 67 males and 63 females with a mean age of 68.40 ± 8.39 years old. In the high-risk group, 96 patients were male and 49 females, aged 73.27 ± 8.76 years on average.

### Baseline data

As shown in Table 1, the difference of hypertension, stroke, prior myocardial infarction (MI), diabetes mellitus, renal insufficiency and smoking between the low-to-intermediate risk and high-risk groups was not statistical significance (all *P* > 0.05). There was no significant difference in the cases of previous PCI, coronary artery bypass graft surgery (CABG) and peripheral artery disease (PAD) between the two groups (all *P* > 0.05). However, a mean age of 68.40 ± 8.39 years old in the low-to-intermediate risk group was prominently lower than that with 73.27 ± 8.76 years in the high-risk group (*P* < 0.001). The gender proportion between the low-to-intermediate risk and high-risk groups was also significant difference, as indicated with 67 male patients in the low-to-intermediate risk group and 96 male patients in the high-risk group (*P* = 0.013). Moreover, the incidence of chronic obstructive pulmonary disease (COPD) was higher in the high-risk group than in the low-to intermediate risk group (*P* = 0.002). Additionally, the LVEF in the low-to-intermediate risk group was surveyed as 66.36 ± 7.09, notably higher compared with 47.09 ± 11.32 in the high-risk group (*P* < 0.001).

**Table 1:** Comparison of baseline data between two groups

Variables	Low-to-intermediate risk group (n = 130)	High-risk group (n = 145)	*P* value
Age (year)	68.40 ± 8.39	73.27 ± 8.76	< 0.001
Male [n(%)]	67 (51.5)	96 (66.2)	0.013
LVEF	66.36 ± 7.09	47.09 ± 11.32	< 0.001
Hypertension [n(%)]	86 (66.2)	95 (65.5)	0.912
Stroke [n(%)]	21 (16.2)	34 (23.4)	0.131
Prior Myocardial infarction [n(%)]	5 (3.8)	7 (4.8)	0.691
Previous PCI [n(%)]	42 (32.3)	47 (32.4)	0.985
Previous CABG [n(%)]	1 (0.8)	0 (0.0)	0.290
PAD [n(%)]	2 (1.5)	4 (2.8)	0.489
COPD [n(%)]	5 (3.8)	22 (15.2)	0.002
Diabetes mellitus [n(%)]	47 (36.2)	45 (31.0)	0.369
Renal insufficiency	3 (18.8)	12(29.6)	0.300
Smoking [n(%)]	20 (15.4)	34 (23.4)	0.093

*LVEF* left ventricular ejection fraction, *PCI* Percutaneous coronary intervention, *CABG* Coronary artery bypass graft surgery, *PAD* peripheral artery disease, *COPD* chronic obstructive pulmonary disease

### PCI outcomes

As elucidated in Table 2, all the PCI parameters including IRA (*P* = 0.333), IABP (2.3% versus 5.5%, *P* = 0.175), arterial puncture route (*P* = 0.293) and mean stent number (2.50 ± 0.874 vs. 2.64 ± 1.07, *P* = 0.233) did not observably differ between the low-to-intermediate risk group and high-risk group.

**Table 2:** Comparison of PCI parameters between two groups

Variables	Low-to-intermediate risk group (n = 130)	High-risk group (n = 145)	*P* value
IRA [n(%)]			0.333
LM	13 (10.0)	8 (5.6)	
LAD	83 (63.8)	105 (72.9)	
LCX	7 (5.4)	5 (3.5)	
RCA	27 (20.8)	26 (18.1)	
IABP [n(%)]	3 (2.3)	8(5.5)	0.175
Arterial puncture route			0.293
Radial artery	115 (88.5)	119 (82.1)	
Brachial artery	7(5.4)	10 (6.9)	
Femoral artery	8 (6.2)	16 (11.0)	
Mean stent number	2.50 ± 0.874	2.64 ± 1.07	0.233

*PCI* percutaneous coronary intervention, *IRA* infarct related artery, *LM* left main coronary artery, *LAD* left anterior descending oronary artery, *LCX* left circumflex, *RCA* right coronary artery, *IABP* intra-aortic balloon pump

### Clinical outcomes

Table 3 presents the clinical outcomes within 30 days and 1 year after PCI. There was no significant difference in stroke, bleeding events (BARC 3 to 5), and incidence of MI within 30 days and 1 year after PCI between the low-to-intermediate risk and high-risk groups. Compared to the low-to-intermediate risk group, patients in the high-risk group had higher cardiac mortality within 30 days after PCI (*P* = 0.019), while no favorable outcome was found in cardiac mortality within 1 year after PCI between the two groups.

**Table 3:** Comparison of clinical outcomes between two groups

Clinical Event [n (%)]	Low-to-intermediate risk group (n = 130)	High-risk group (n = 145)	*P* value	Low-to-intermediate risk group (n = 130)	High-risk group (n = 145)	*P* value
	Within 30 days after PCI			Within 1 year after PCI		
MACE	4 (3.1)	9 (6.2)	0.222	17 (13.1)	19( 13.1)	0.995
Cardiac death	0 (21.3)	6 (4.1)	0.019	3 (2.3)	9 (6.2)	0.114
MI	3 (2.3)	2 (1.4)	0.565	6 (4.6)	6 (4.1)	0.847
TVR	2 (1.5)	1 (0.7)	0.499	10 (7.7)	6 (4.1)	0.209
Stroke	1 (0.8)	1 (0.7)	0.938	1 (0.8)	2 (1.4)	0.627
Bleeding*	3 (2.3)	5 (3.4)	0.574	10 (7.7)	10 (6.9)	0.800

*MACE* major adverse cardiac events, *MI* myocardial infarction, *TVR* target vessel revascularization

*Bleeding events with Bleeding Academic Research Consortium (BARC) 3–5

### ROC curve analysis

**Fig. 1:**
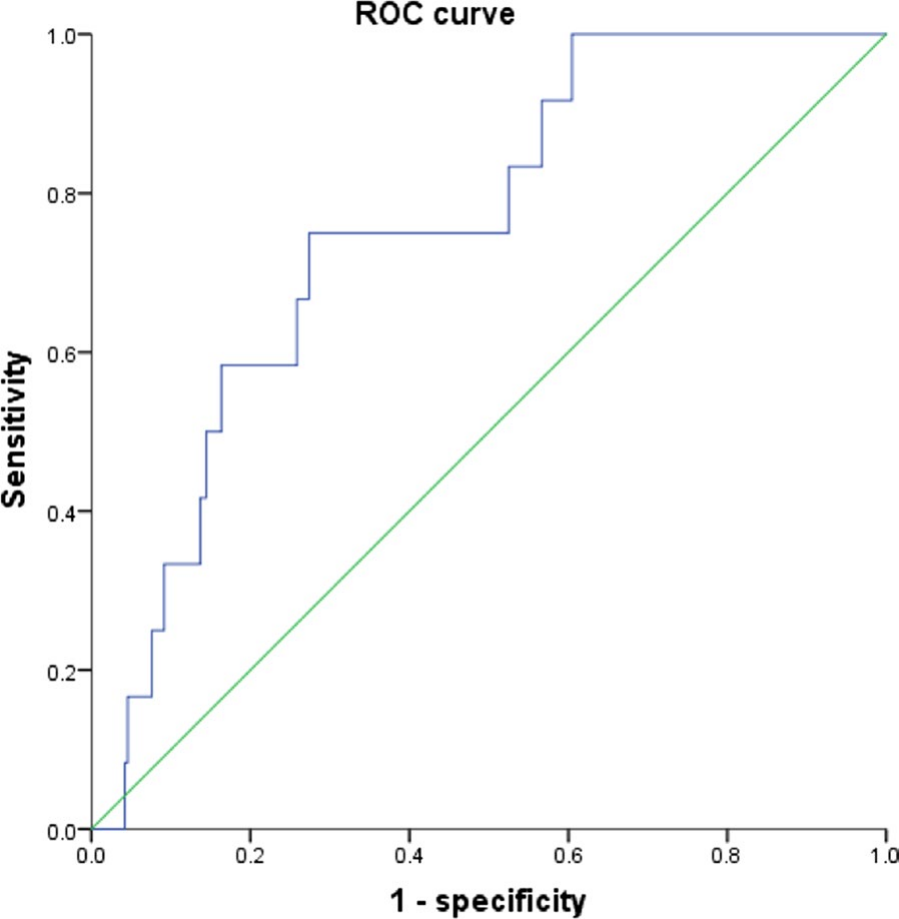
ROC curve of the ACEF scoring system in predicting cardiac death at 1 year after PCI. ACEF, age, creatinine and ejection fraction; RA, rotational atherectomy; PCI, percutaneous coronary intervention

The area under the ROC curve of the ACEF scoring system in predicting cardiac death at 1 year after PCI was calculated as 0.756 (*P* = 0.003), as exhibited in Fig. 1. Additionally, the sensitivity and specificity of the ACEF scoring system in predicting cardiac death at 1 year after PCI was 75.0% and 72.6%, respectively.

**Fig. 2:**
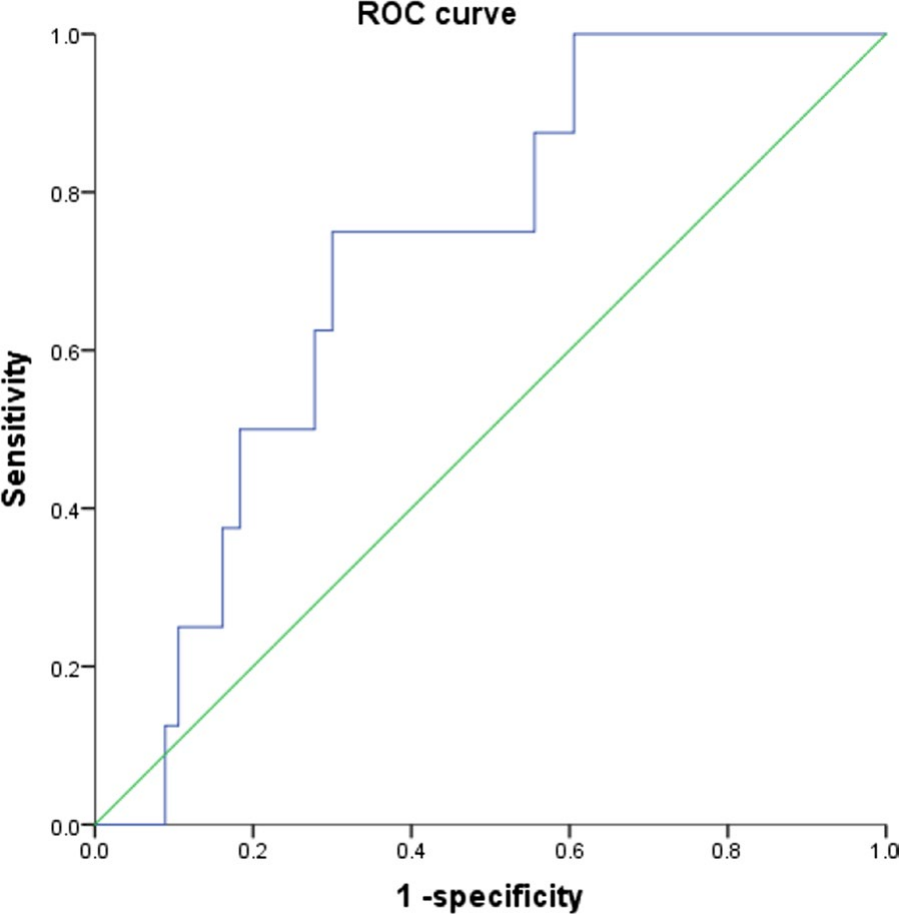
ROC curve of the ACEF scoring system in predicting cardiac death at 1 year after LAD-PCI. ACEF, age, creatinine and ejection fraction; LAD, left anterior descending oronary artery; PCI, percutaneous coronary intervention

Furthermore, the area under the ROC curve of the ACEF scoring system in predicting cardiac death at 1 year after LAD-PCI was calculated as 0.715 (*P* = 0.04), as illustrated in Fig. 2. Additionally, the sensitivity and specificity of the ACEF scoring system in predicting cardiac death at 1 year after LAD-PCI was 75.0% and 70.0%, respectively.

## Discussion

With the increase of age, coronary atherosclerosis in patients with coronary heart disease is dynamically evolving. The plaques develop and begin to appear calcified plaques, and smooth muscle cells and foam cells begin to mineralize, which promotes the deposition of calcium salts in the plaques to cause calcification. Rotational atherectomy has been demonstrated for the preparation of undilatable lesion including heavily calcified coronary lesions to implant the stent, thereby improving procedural success of PCI. Therefore, an accurate risk assessment based on the overall rate of MACE is very imperative and significant due to clinical needs.

The variety of risk-assessment models have been used for risk stratification of the patients with PCI. For instance, EUROScore and modified EUROScore have been constructed for patients undergoing PCI [15, 16]. SYNTAX score has a predictive value for unprotected left-main coronary artery (uLMCA) patients with PCI [17]. However, the SYNTAX score is notably involved in worse three-year outcomes [17]. In addition, the Clinical SYNTAX score (CSS) also has been reported to show a predictive value in patients with heavily calcified coronary stenosis undergoing RA with stent implantation [11]. The ACEF score, containing three core parameters of age, creatinine and LVEF, is simple and handy risk- stratification model for PCI, which has been confirmed by our team in STEMI patients aged ≥ 75 years old [10], as well as other groups [8, 18, 19]. The age, gender proportion and LVEF between the low-to-intermediate risk and high-risk groups was significant difference, but the PCI parameters between the two groups had no statistical difference, which suggested the application of ACEF score between the two groups in the present study. Furthermore, the predictive value of ACEF score for clinical prognosis of patients diagnosed with heavily calcified coronary lesions after PCI with RA was assessed. The ROC curve analysis revealed that the ACEF score could effectively differentiate the short-term mortality rate of 275 incorporated patients diagnosed with heavily calcified coronary lesions and have predictive value, which was accordance with previous study reported that ACEF score can predict MACE in patients diagnosed with heavily calcified coronary stenosis at 1 year after RA with stent implantation [11].

However, there are still some limitations that need to further improve. For example, the duration time of the follow-up is 1 year, the sample size is relatively small, and all patients incorporated in the present study are from a single center. Therefore, results of extended duration of follow-up with bigger sample size from multiple centers can underpin the conclusion shown in the present study.

## Conclusions

ACEF value upon admission can predict the cardiac death rate at 1 year after PCI with RA in heavily calcified coronary lesions patients.

## Data Availability

Not applicable.

## Abbreviations

ACEF: Age, creatinine, and ejection fraction
PCI: Percutaneous coronary intervention
RA: Rotational atherectomy
LVEF: Left ventricular ejection fraction
MACE: Major adverse cardiovascular events
ACT: Activated coagulation time
ISR: Revascularization, stent restenosis
SD: Standard deviation
uLMCA: Unprotected left-main coronary artery
CSS: Clinical SYNTAX score
